# More‐than‐care: People with intellectual disability and emerging vulnerability during pandemic lockdown

**DOI:** 10.1111/tran.12595

**Published:** 2023-01-11

**Authors:** Ellen van Holstein, Ilan Wiesel, Christine Bigby, Brendan Gleeson

**Affiliations:** ^1^ RMIT Melbourne Victoria Australia; ^2^ School of Geography, Earth and Atmospheric Sciences University of Melbourne Carlton Victoria Australia; ^3^ School of Allied Health, Human Services and Sport La Trobe University Melbourne Victoria Australia; ^4^ School of Design University of Melbourne Melbourne Victoria Australia

**Keywords:** cognitive impairment, coronavirus, emergency response, exclusion, NDIS, risk

## Abstract

This paper offers more‐than‐care as a framework for analysing how vulnerability emerges in the lives of people with intellectual disability beyond relations of care. More‐than‐care detaches vulnerability from the identity category of disability. It provides a framework for conceptualising vulnerability in an unequal, neoliberalising, and ableist world and sheds new light on the ever‐evolving constitution of vulnerability and disability. This intervention breaks with conceptions of vulnerability centred on care needs that leave other circumstances that inform vulnerabilities unexamined. Importantly, the framework shifts responsibility for managing vulnerabilities away from carers alone. The more‐than‐care framework is grounded in socio‐material conceptualisations of disability and advances a tripartite framing of vulnerability. First, it grounds studies of vulnerability in histories of spatially uneven investment in infrastructure and resources that shape how care and other practices can assemble to produce, challenge, and manage vulnerability. Second, it recalibrates dominant conceptions of the temporality of vulnerability to ensure sensitivity to the unpredictability of emergent vulnerabilities. Third, in following a socio‐material conceptualisation of intellectual disability, more‐than‐care expands discussions about agency in the context of vulnerability. These concepts are empirically examined through an analysis of how vulnerability emerges in the lives of four self‐advocates with intellectual disability during Melbourne's first and second COVID‐19 lockdowns. The analysis shows that vulnerability was highly dynamic and unpredictable as it emerged in complex socio‐material assemblages that included care arrangements, embodied experiences and agencies, and past instances of neglect and exploitation.

## Short Abstract

This paper offers more‐than‐care as a framework for analysing how vulnerability emerges in the lives of people with intellectual disability beyond relations of care. The framework breaks with conceptions of vulnerability centred on care needs that leave other circumstances that inform vulnerabilities unexamined. Applying the framework to the emergence of vulnerability in the lives of self‐advocates with intellectual disability during Melbourne's COVID‐19 lockdowns showed that vulnerability was highly dynamic and that the self‐advocates actively managed emerging vulnerabilities.

## 1 INTRODUCTION

Geographers interested in risk reduction follow definitions that understand vulnerability to consist of someone's exposure to risk combined with their ability to cope with impacts (e.g., Adger, 2006). As a result, vulnerability due to disruptive events is commonly understood as knowable and preventable, provided people are offered support and care that help them cope. This understanding is frequently applied to people with disability and consequently, recent efforts to reduce the vulnerability of people with disability in disruptive events have created a disproportional focus on care – narrowly understood in terms of support services – in vulnerability prevention (Ton et al., 2019). This focus is out of step with recent developments in disability studies, where disability has become understood as an emergent and therefore highly unpredictable phenomenon (Cluley et al., 2020) and where care is regarded with suspicion for the asymmetrical power relationships it creates and for its potential to entrench dependency (Clough, 2021; Hall, 2011; Wiles, 2011).

Geographers writing on care from the perspective of feminist care ethics understand vulnerability as a universal condition, and care as a broad concept that concerns all practices that sustain life through webs of interdependency. This includes a broad array of infrastructures that are not traditionally associated with care provision, such as housing and food systems, which enable caring practices (Power & Mee, 2020; Williams, 2016). However, for people with intellectual disability and other groups who are commonly perceived as ‘vulnerable’, this tendency to broaden interpretations of vulnerability and care generates new risks. People with intellectual disability are commonly regarded as especially vulnerable and, as a result, all their relationships, including with people who are not their carers and would not be suitable as carers, are often interpreted as relationships of care, in a narrower and more one‐sided sense (for a recent discussion, see Power & Bartlett, 2019). With this, many aspects of their lives become about their disability and support needs. Conceptualising vulnerability as an unmet need for care thus perpetuates dominant tendencies to essentialise a person's disability and risks overlooking vast dimensions of the lifeworlds of people with intellectual disability, including their strategies for coping with vulnerability that fall beyond the provision of disability support.

This paper develops a more‐than‐care framework to guide socio‐material analyses of vulnerability in the lives of people who are labelled ‘vulnerable’. Drawing on assemblage theories of intellectual disability (Cluley et al., 2020; Feely, 2016) and on the conceptualisation of vulnerability as an emergent effect of assemblages (Webber, 2013), more‐than‐care develops an analytical framework of vulnerability that is spatially sensitive, attends to the histories of embodied experiences of intellectual disability, and foregrounds the agency of people with intellectual disability to respond to emergent vulnerabilities. The conceptual approach to vulnerability as more‐than‐care is explored through the experiences of people with intellectual disability who managed pre‐established and new vulnerabilities during the 2020 COVID‐19 lockdowns.

Lockdowns subjected Melbourne's metropolitan region of five million inhabitants to a set of far‐reaching and disruptive interventions, including a restriction of non‐essential travel to a five‐kilometre radius, a one‐hour per day limit on time spent outside the home, and a strict 8 pm to 5 am curfew confining people inside at night. Lockdowns were presented as collective risk management, with state politicians evoking a discourse of ‘being‐in‐this‐together’. However, the impacts of the lockdown were unevenly distributed and felt hardest by those deemed ‘vulnerable’, due to additional interventions that targeted people with underlying health risks and due to prejudice in decision‐making. Some decisions aimed to protect so‐called ‘vulnerable’ people from the risk of COVID‐19 but exposed them to other harms. For example, residents of public housing towers were suddenly forced into a highly contested ‘hard lockdown’ that was police‐enforced and harsher than interventions in other buildings (Young, 2021). Likewise, the few infections that spread into group homes of people with disability led all homes to preventatively refuse visitors, isolating residents from family and friends (Kinsella & Campanella, 2020). Such interventions meant that people with disability experienced greater inconvenience and isolation. While lockdowns were an important public health measure that provided opportunity to ensure medical care was available for those infected during the first stages of the pandemic, Melburnians bore the emotional and material burden of isolation and restrictions unevenly (Young, 2021).

We draw on a set of interviews with four self‐advocates with intellectual disability that were conducted during Melbourne's 2020 lockdowns. These interviews were part of a larger three‐year study (2018–2021) that analysed the accessibility of mainstream services for people with intellectual disability. We developed close collaborative relationships with four research advisers with intellectual disability as part of this work. Ongoing conversations with these advisers over Melbourne's two 2020 lockdown periods created invaluable insight into the stress that unclear communication of risk and ever‐changing restrictions caused people with intellectual disability. Furthermore, the ongoing nature of these conversations provided unique insight into how people with mild intellectual disability who live independently and who have personalised funding for disability support experienced interruption of routines, confinement at home, and the emergence of new access barriers to services.

We contend that addressing vulnerability requires an analytical framework of more‐than‐care, that is, a framework that scrutinises how vulnerability emerges in place and over time through a wide variety of relationships and material circumstances, including relationships of care. The more‐than‐care lens offers geographers rich insight into how vulnerability emerges at times of disruption and demonstrates that emerging vulnerabilities were the result of lacking care, restrictive care (i.e., a social system of support that does not prioritise the rights of people with intellectual disability to make decisions about their own lives), and a suite of other conditions, such as inadequate information, that impede people's ability to manage emerging vulnerabilities. In developing this framework, we follow Fisher and Tronto's definition of care as a ‘species activity that includes everything that we do to maintain, continue, and repair our “world” so that we can live in it as well as possible. That world includes our bodies, our selves, and our environment, all of which we seek to interweave in a complex, life‐sustaining web’ (1990, p. 40). Tronto has defined care as being performed through practices of caring about, taking care of, care giving, and care receiving (Tronto, 1993), and later added ‘caring with’ to acknowledge an ethic and practice of care that align with the democratic values of solidarity, justice, and equality (Tronto, 2013). ‘Caring with’ cannot be an isolated act between a giver and receiver of care, but rather it is a practice that unfolds with spatialities, temporalities, and materialities that shape caring capacities (Power, 2019). Tronto and followers' care ethics offer comprehensive and nuanced ways of thinking about care that acknowledge the complex power relationships between care givers and receivers. Yet, we argue that to ensure that the diverse sources of vulnerability and the agency of people deemed ‘vulnerable’ remain visible, it is important to analyse vulnerability during disruptive events as emanating from more‐than‐care.

To develop the more‐than‐care framework, the rest of the paper follows in five parts. Section 2 discusses socio‐material theories of disability and vulnerability. Section 3 introduces the more‐than‐care framework. Section 4 sets out the research context and methods. Section 5 applies a more‐than‐care lens to the self‐advocates' accounts to analyse their experiences of and strategies for managing vulnerabilities. To conclude, we discuss the implications of a more‐than‐care approach in geographical studies of vulnerability. Specifically, we expand on the potential for this framework to further decentre disability and other identity categories as sources of vulnerability and to raise urgent questions about the distribution of responsibility for the management of vulnerabilities.

## 2 SOCIO‐MATERIAL THEORIES OF DISABILITY AND VULNERABILITY

Assemblage theory has enabled social scientists to create ‘a process‐based ontology of movement in which the world is conceived of as a dynamic and open‐ended set of relational transformations’ (McCormack, 2009, p. 277). This ontological turn has drastically changed scholarship on both disability (Feely, 2016; Hall & Wilton, 2017) and vulnerability (Harrison, 2008; Webber, 2013). In disability studies, assemblage theory offered a response to growing critique of the social model of disability. While many disability scholars and advocates embraced the social model as a replacement of its pathologising predecessor – the medical model of disability – critics pointed out that the social model's emphasis on disabling environmental and social barriers downplays the importance of embodied impairments (Wiesel & van Holstein, 2020). Assemblage theory has proven useful as it informed an understanding of disability as co‐constituted through multiple (bodily, cognitive, environmental, discursive, economic) changing circumstances in someone's life and it is therefore better able to attend to the dynamic nature of living with disability (Cluley et al., 2020). In comparison to the social model's emphasis on structural barriers, this attention to fluidity and dynamism also offers an empowering perspective because it recognises the potential for change and resistance that is embedded in everyday interactions and practices (Wiesel & van Holstein, 2020). This shift offers important new ways for understanding the agency of people with intellectual disability.

Efforts to include people with disability in disaster risk reduction so far tend to build on dominant conceptualisations of disability, such as the social model of disability, that have been less than perfectly suited to represent the experiences of people with intellectual disability. As argued by Cluley et al. (2020), the social model of disability, as well as feminist and phenomenological theorisations, are implicitly underpinned by a mind–body dualism that pre‐supposes that a person with disability is cognitively able to recognise their needs and interests, to autonomously choose appropriate goods and services, and to collectively organise with peers toward shared objectives, even when this is not true for all people with intellectual disability. In disaster reduction research, these dominant theorisations of disability have found expression in an emphasis on participation in disaster preparedness as a central means to reduce the vulnerability of people with disability (Ton et al., 2019). In this context, vulnerability is framed as a gap between a person's disability support needs and the provision of care, narrowly understood in terms of meeting such support needs. Vulnerability thus becomes understood as a phenomenon that can be rationally measured, predicted, and prevented through the provision of care, if only a person with disability anticipates their needs.

An approach to vulnerability as a knowable care gap overlooks the dynamic nature of disability and vulnerability, it conceptualises disabled bodies as cognitively able, and it underestimates the ongoing disempowering stigma of requiring care that many people with disability regularly face, which makes many prefer not to discuss their lives in terms of vulnerability and care (Clough, 2017, 2021; Scully, 2013). Many people with disability and disability scholars prefer to emphasise that vulnerability is a universal condition and stress the dignity of risk as a form of self‐determination (Wiesel et al., 2021). Recent interventions in policy and legislation in countries around the world reflect this shift from a view of people with intellectual disability as ‘vulnerable’ to an emphasis on self‐determination, for example through the introduction of personalised budgets that aim to give people with disability choice and control over the support they receive. While offering many people with disability new opportunities and decision‐making power, such systems have also been critiqued for exposing some people to new risks of neglect and exploitation (David & West, 2017; Hall, 2011). Concurrently, scholars in disability studies have pointed out that a universal framing of vulnerability may stifle empirical research into the particular conditions that make some people more vulnerable than others and that this makes it harder to improve those conditions (Scully, 2013).

The socio‐material approach that has reshaped understandings of disablement has also been adopted to rearticulate theories of vulnerability in ways that makes those theories better attuned to the changing circumstances – such as relationships, events, and embodied experiences – that underpin vulnerability. Working with people from Kiribati who were labelled as extremely vulnerable to climate change, Webber (2013) identified that their vulnerability was an emergent effect produced by a socio‐material assemblage consisting of climate change and coastal erosion but also traditional customs, international aid relationships, performative practices of vulnerability, and more. Such a conceptualisation of vulnerability is attuned to the changing nature of vulnerability and offers important potential for analysing vulnerability in ways that do not essentialise a person's impairment, care needs, or broader biological traits. We use these socio‐material understandings of disability and vulnerability to develop a more‐than‐care approach to understanding the emergence of vulnerability.

## 3 FRAMING VULNERABILITY AS MORE‐THAN‐CARE

In this section we advance a socio‐material conceptualisation of vulnerability that can help explain how vulnerability emerges and how people manage it beyond the remit of care. We label this approach more‐than‐care as it considers how vulnerability emerges through a wide set of circumstances and practices, including care, but without taking an a priori focus on care. The framework understands vulnerability as ‘being more than usually likely to experience the bad things that can happen to humans’ (Scully 2013, p. 210). This definition recognises the universal experience of vulnerability, while also attending to individual support needs and the uneven ways support is institutionally organised, including the social organisation of privilege and disadvantage that shapes an individual's ever‐changing capability to protect themselves against harm (Fineman, 2010). This approach aligns with socio‐material conceptualisations in which, ‘the [intellectually] disabled body is materially present in a non‐essentialist relationship with a multitude of other factors, phenomena, experiences and discourses, all of which serve to produce the lived experience of [intellectual] disability’ (Cluley et al., 2020, p. 248). More‐than‐care enables an analysis of vulnerability that recognises a wide set of practices and circumstances that inform lived experiences of vulnerability and that afford people with disability opportunity to articulate what constitutes care or a care need and what lies beyond. The more‐than‐care framework draws together three considerations around care and vulnerability.

First, more‐than‐care includes care and builds on research literature that analyses how care is practised in the context of spatially uneven investment in care infrastructure that shapes capacities to care (Power, 2019). Geographers in this field explore how care emerges through assemblages of a broad range of human and non‐human actors engaged, however unevenly, in a wide array of life‐sustaining practices. Concurrently, geographers recognise that some threats to prospering and survival are better addressed by considering not only ‘care’ but also ‘justice’, or care‐full justice (Williams, 2016, 2017). This opens up consideration of distributive inequalities and recognition in parallel to analyses of care, acknowledging the two are deeply intertwined. Incorporating these insights enables the more‐than‐care framework to be sensitive to structural injustices and inequalities that underpin ableist discrimination and the uneven availability of essential infrastructures and services – such as secure access to housing, eligibility for welfare and support payments, and access to affordable transport options – and how these can trigger, soften, and deepen vulnerabilities.

Second, more‐than‐care challenges dominant conceptions of the temporality of vulnerability. When vulnerability is framed as a preventable future condition, people's circumstances in the present are normalised and become entrenched as a benchmark to which to return after disruption. Furthermore, vulnerability is then understood as a care need that emerges as the consequence of an isolated disruptive event. Conversely, when vulnerability is understood as an emergent effect in a constant state of becoming (Webber, 2013), the many dimensions of a person's life that have fed into the manifestation of vulnerability over extended periods of time become a key analytical focus (Anderson et al., 2020; Mould et al., 2022). Rather than drawing temporal boundaries around instances of vulnerability, more‐than‐care acknowledges the blurred lines between normality and crisis that people experience as they are affected by combinations of crises of different intensities and durations (Anderson et al., 2020; Berlant, 2011). In opening up considerations of the temporal dimensions of vulnerability, more‐than‐care offers a useful lens for thinking about people with intellectual disability's ongoing experience of ableist exclusion from mainstream community settings and how these shape expectations and experiences of vulnerability during disruptive events. This way, more‐than‐care embeds vulnerability in contexts of people's everyday practices and experiences without conceptualising the entirety of people with disability's lifeworld as evolving around their need for care.

Third, in following a socio‐material conceptualisation of intellectual disability, more‐than‐care expands discussions about agency in the context of vulnerability. Work on disaster risk reduction for people with disability recognises that some forms of agency are achieved in ways that go beyond the individual, but because it understands agency foremost as an individual characteristic, it frames distributed forms of agency as external to the person and as depending ‘on an individual's informal relationship with other people and the willingness of those who provide or create such capabilities’ (Ton et al., 2019, p. 14). Such a direct connection between disability and dependency is dangerous when labels of ‘dependency’ are persistently used to patronise, segregate, and silence people with intellectual disability (Clough, 2017). Following a socio‐material conceptualisation of disability, more‐than‐care adopts the idea of ‘co‐corporeality, where bodies are not just contiguous and mutually self‐reliant but entwined with one‐another’ (Cluley et al., 2020, p. 247). Feminist care literature challenges ‘dependency’ by emphasising inter‐dependency, and challenges one‐directional care by offering a more democratic and reciprocal ‘caring with’. In interpreting some practices related to vulnerability as outside of care, a more‐than‐care lens builds on this work by foregrounding the collaboratively and individually achieved agency of people with intellectual disability and it positions people with intellectual disability as active agents of change through interdependency and collective action.

## 4 RESEARCHING PANDEMIC LOCKDOWN

**FIGURE 1:**
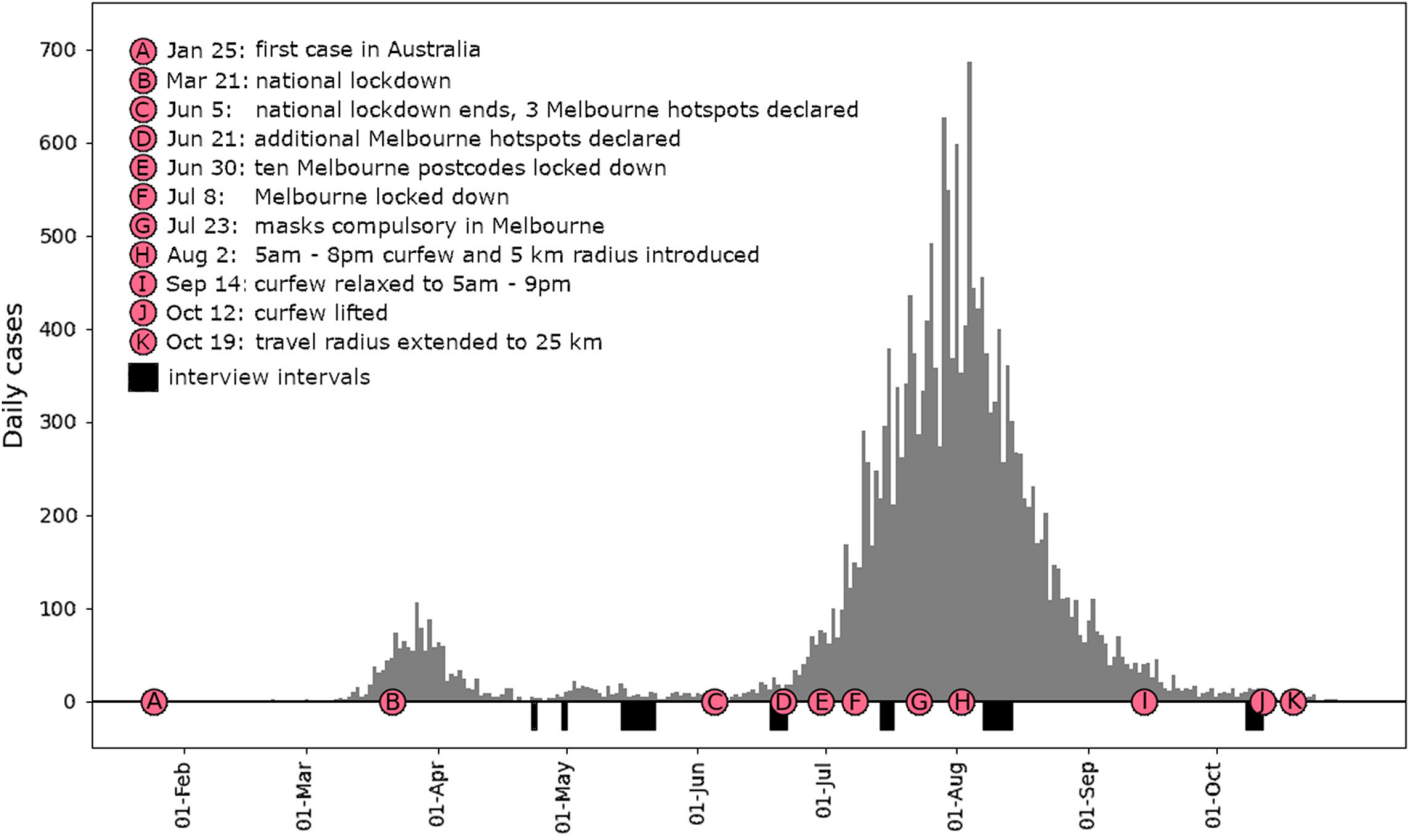
Timeline of daily cases, restrictions, and interview intervals

In 2020, the metropolitan region of Melbourne was subject to one of the most extensive responses to COVID‐19 outbreaks in the world. Seeing daily cases in Victoria surge to 106 during the first wave and to 723 in the second, the State Government designed a far‐reaching program of restrictions and policing to impose, among other things, mask‐wearing, limitations on leaving the home, and the closure of non‐essential services (Figure 1). These measures had profound impacts on everyone in Melbourne. Impacts were also felt by people with intellectual disability, for whom disruptions interacted with support needs and how their support had been organised.

Australia has seen a complete overhaul of disability support funding, with the introduction of personal budgets for eligible people with disability as part of the National Disability Insurance Scheme (NDIS) (Wiesel et al., 2019). Since its roll‐out in 2013, the NDIS has created a mushrooming of businesses that provide disability support and has led to the further casualisation of what was already a precarious workforce. The NDIS was welcomed for its promises to double public expenditure on disability support and to place people with disability in control of purchasing supports. However, the NDIS has also received fierce critique as it favours people who have articulate advocates, erodes collective forms of activism, and fails to protect many people with disability against abuse and neglect (e.g., David & West, 2017). Initial observations from the COVID‐19 pandemic revealed some of the vulnerabilities that have been created by relying on casualised and underpaid staff for the provision of disability support, as disability researchers identified the risk of support workers showing up to work while unwell or not presenting to work without organising replacement (Dickinson et al., 2020).

This paper is informed by research on the accessibility of mainstream services for people with intellectual disability in four Australian cities. A reference committee consisting of four Melbourne‐based self‐advocates with intellectual disability sits at the centre of our enquiry. Before restrictions, the group met quarterly to advise and comment on research design, data interpretation, and dissemination. From February 2020, meetings continued one‐on‐one via teleconferencing and focused on the impacts of the pandemic on advisers' lives, their use of mainstream services, and their strategies around interpreting and implementing changing public health settings.

The self‐advocates had some circumstances in common. All four had individual support funding through the NDIS and all worked for disability rights organisations. Because of their experience in advocacy, they were all skilled and relatively confident in articulating their experiences and support needs. For these reasons, they do not represent the diverse population of people with intellectual disability. People with more severe disability and people who were less practised in speaking up were affected in different ways. Beyond their shared skills and experiences, the advocates' contexts differed. For instance, while Greg and Luke lived at the same address as their parents, Sue and Heather did not. Greg and Heather lived in parts of the city that were repeatedly declared a COVID‐19 hotspot and they thus had to weigh additional factors when navigating risk and rules during the two lockdowns.

The paper draws on a total of 28 interviews with the self‐advocates. Between April and October, each of them participated in seven interviews that lasted between 20 and 50 minutes. The shift to one‐on‐one interviews rather than group tele‐conferencing ensured that the advisers continued to have equal opportunity to participate. Interviews were held as soon as advisers were available after the easing or tightening of restrictions and as a result periods between interviews ranged from one to five weeks (Figure 1). Heather was called away during her third interview, and this interview was resumed later that week. The research advisers were paid for contributing their time and expertise. After several conversations with the self‐advocates about privacy and recognition, the self‐advocates requested to be identified by their first names.

Interviews were analysed thematically using the steps set out by Braun and Clarke (2006). After each round of interviews the authors discussed the data and noted down early impressions. When all interviews were conducted and transcribed, descriptive codes such as ‘routines’, ‘information provision’, and ‘formal and informal support’ were identified, which were subsequently used to inform the articulation of broader explanatory themes such as ‘agency/managing vulnerability’, ‘spatial constraints’, and ‘residential context’. Analysis focused on identifying sources of vulnerability and this allowed for understanding people with intellectual disability as managers, rather than mere subjects of vulnerability. In what follows, we analyse how vulnerability emerged through more‐than‐care to deepen understanding of how vulnerability is socially and politically produced.

## 5 EMERGING VULNERABILITIES OF PANDEMIC LOCKDOWN

### 5.1 Vulnerability and the organisation of care

The self‐advocates demonstrated initiative and efficacy in managing their formal and informal supports during lockdowns. Their position as procurer of support, facilitated by NDIS personalised funding, gave the self‐advocates control over how support was provided. Sue said about support workers who came to her house:

I make sure that they cancel if they are feeling any symptoms, even if they think it's a cold, I say, look, I'd rather you cancel, because I just don't want to take the risk of it being just a cold, and it then being coronavirus. (Fourth interview, 22 June)

Luke also described a cautious approach, deliberating with peers to best manage his support workers in a way that minimised risk of exposure:

I've been speaking to a lot of friends also that have done it, and I decided off my own bat to limit how many people were coming into my house which a couple of carers are a bit – they were understanding, but they go, well, does that mean we lost you, we lost the work? I said no, you haven't lost me as a client, but until this is over, however long that is, there's only going to be two staff, three staff maximum. (First interview, 23 April)

Sue and Luke's proactive approach to managing their support highlights that for some people with intellectual disability who were part of self‐advocacy networks and who were independently or collaboratively able to identify risk, personalised budgets were empowering, including in the context of a pandemic.

The stories indicate that the vulnerabilities of people with disability and those of their paid support workers become closely entwined when they rely on each other for sustenance and for minimising risk of viral infection. Sue's quote below shows how decision‐making about managing exposure to the virus is shared with support workers who are asked for input into some of these decisions:

Ellen Do you use a mask?

Sue I do when I'm out shopping and sometimes I ask my carers if they would like me to wear a mask, if they want me to, I will, but none of them have wanted me to yet. (Fifth interview, 17 July)

The control that comes with personalised budgets creates responsibilities for which people with intellectual disability receive limited additional support (Hall, 2011). This creates situations in which a paid support worker can be a main source of risk but also the key person to assist with identifying and managing risk. Sue and Luke's approaches demonstrate that this means they have to navigate complicated conflicts of interest as they adjust to restrictions and emerging vulnerabilities. In distinguishing between practices of formal care provision and practices of managing vulnerabilities in the face of that care, more‐than‐care highlights the agency and efficacy of people with intellectual disability.

Throughout the pandemic, there was concern that some disability support organisations might cease to provide support to clients, for example when staff were unwell, self‐isolating, or because they were no longer allowed to work across multiple sites (Dickinson et al., 2020). Uncertainty about support provision had different impacts on the self‐advocates. Luke, for example, lives in a house on the same block of land as his parents and he felt assured that they would step in as needed:

If I couldn't get a support worker, god forbid. But my parents are still fit enough to help me around the house. But I don't – it's funny. I say to my – a lot of my NDIS planners that I've had, I say to them, well, my mum and dad are here for retirement, they're not here to help me. But I do know that if worse came to worst, I do know that, without a doubt, if I asked my mum or my dad to help me, without a doubt, they'd just jump straight in and do what has to be done. (First interview, 23 April)

Luke's experience demonstrates the importance of trusted providers of informal support at times of emergency and uncertainty (Connon & Hall, 2021). His account also illustrates that personalised support enables him to shield his relationship with his parents from being defined as indissolubly tied to his care needs.

While some people were tasked with managing supportive relationships within the changing rules of restrictions, others were tasked with managing the absence of support. Heather did not have supportive family members around and she experienced a six‐week gap in support provision when her regular support worker took leave without arranging replacement:

I rang up the company and I asked for another support worker, and they said that isn't how they worked. And I just told them, I'm not getting any support at all. Then they said they will try and see what they can do, but they can't promise anything. (Third interview, 22 May)

Heather's experience also demonstrates that the purported control personalised funding provides over contracted support may be overstated, especially when necessary support is not available at a time of disruption. Incidentally, this hiatus occurred when Heather's suburb was declared a hotspot. At this time, Heather also learned that she had been exposed to the virus, creating a new need for support with accessing unfamiliar services and interpreting complex information.

The interruption of paid support forced Heather to rely on the goodwill of informal providers of support in her social networks. Heather lived with her husband who has a cognitive impairment. Because he had also been exposed and because the hospital initially refused to test them due to lack of symptoms, Heather proactively engaged a general practitioner and a counsellor to subsequently tag along with her partner and his worker to gain access to testing:

Ellen Has anyone been in touch with you about what happened at [the exposure site]?

Heather I got in contact with my – the workers from my volunteer work. […] I told them what actually happened, and they said that they'll – if I let them know they can help me if they can. I've also got my husband's support worker who's going to help get a test done. (Third interview, 18 May)

People with intellectual disability are often forced to rely on advocates to negotiate access to mainstream services (Bigby & Fyfe., 2012) and Heather's story illustrates that this continued during lockdown when some people's access to advocates was interrupted. Such access barriers limit people with intellectual disability's opportunities to manage emerging vulnerabilities. In this case, Heather managed to access the required services using her partner's support, but this was fortuitous as the same providers of support also limited her ability to cope with lockdown restrictions. For example, Heather reported that her husband's support workers were unable to include items for her when they visited the supermarket to shop for him, and they denied them the opportunity to take out a household internet connection that would have reduced both their costs, because they were concerned that Heather might use more than half of the data. These dynamics illustrate that providers of disability support continue to work with highly individualised conceptions of care needs and overlook how the management of vulnerability is achieved in collaborative, networked, and reciprocal ways. This shows that vulnerability is not the mere opposite of agency or ‘negative care’ (Milligan & Wiles, 2010) because care practices create vulnerabilities when they limit people's control over decisions and because people with intellectual disability manage vulnerabilities with and despite disability support.

### 5.2 Understanding changing circumstances

During the pandemic lockdowns, up‐to‐date information about case numbers, restrictions on social gatherings, and limits on travel radiuses was an essential resource for managing vulnerability. Sue explains how she concluded where to visit and avoid, based on her assessment of risk:

I just don't feel like going [to the CBD], because for me to get there, one of the […] spots was Darebin Council, which I'd have to go through on the train to get to the city. […] How do I know that no one's getting on my carriage, from, let's say, Fairfield, which Fairfield is in Darebin, doesn't have the coronavirus? (Fourth interview, 22 June)

Sue illustrates the importance of understanding up‐to‐date information and the complex task involved in translating this information into a mental map of the city that considers which suburbs are located in which Council area and how these align with trajectories of travel.

The self‐advocates had to extract essential information from media and government messaging that was not tailored to their needs and they reported that they frequently found mainstream news confusing. They often turned to other services and to their personal networks to complement what they heard on the news. We asked Sue what kind of organisations she turned to for suitable information.

I think mainly disability advocacy organisations. [One of them is] in [Tasmania], and they do it really well. But of course, they're only giving out what's – their information might be only good for Tassie, but as I've said in their chats, I think, from what I understand of it, until at least – until recently, it's been pretty much on par between Tassie and Victoria anyway. […] So, sometimes, it's not what the whole country says. But some of it is okay, like the washing your hands and the 1.5 metres, and covering your mouth and them sort of things. (First interview, 24 April)

As pointed out by Sue, this way of accessing the news required an additional understanding of differences between rules in different places to interpret and apply the information successfully.

The interruption in support provision that Heather experienced impacted on her ability to interpret important information, and this made her turn to friends and their support workers to clarify new rules:

It's a bit confusing on the news, because I heard on the news one morning there's only three reasons to be out. It was grocery shopping, medical appointments and work – going to work. It said you weren't allowed to exercise, but they didn't add on – it's only for those who are in isolation, because we've been getting a lot of people going out exercising when they were meant to be isolating. […] I had to ring up a friend and get them to explain it better to me. I forget what I exactly asked, but I just let her know what I heard on the news. She had her support worker with her at that time, so I think that support worker explained to her, and then she passed it on to me. (Sixth interview, 10 August)

Somewhere during the relay of information, rules became misinterpreted as exercise was one of the legitimate reasons to leave the home during lockdowns, but people in isolation were not to leave their homes at all.

Risk of misinterpretation also emerged because generic public health messaging did not consider the particular circumstances of some people with disability. Sue, for instance, reported that she was unsure whether rules about leaving the home applied to her:

If I heard the word exercise, I think they mean walking but when I'm in a wheelchair, I'm not really exercising.

This account demonstrates the compounding effects of the embodiment of intellectual disability with other kinds of disability. In the circumstance of changing restrictions, this created the extra cognitive task of having to translate generic public health messages to specific embodiments. Sue's experience shows how this led to self‐exclusion and it illustrates how emergency response can create uneven vulnerability to isolation.

The lack of clarity in mainstream reporting and the lack of skills of mainstream service and support staff to assist with the translation of information disadvantaged the self‐advocates and frustrated their ability to make effective adjustments to their everyday routines. Inaccessible information added to this group's support needs during lockdowns, and this shows that analyses of vulnerability need to move beyond a focus on care to include broader sets of relationships, such as those with services that provide information that is essential to people's ability to manage vulnerabilities. A more‐than‐care lens highlights that vulnerability emerges as discursive contexts impact differently on disparate embodied and social experiences of living with intellectual disability.

### 5.3 Spatial reconfigurations and the embodiment of intellectual disability

Spatial reconfigurations of metropolitan Melbourne as part of COVID‐19 lockdowns caused disruptions of routines, such as a sudden shift to working from home and an inability to seek assistance in shops. While these were common experiences for many people during the lockdowns, people with intellectual disabilities' smaller social networks and low incomes meant that their challenges to adjust were more intense compared with those of other more affluent and socially connected people. For example, Heather struggled with a limited phone data plan that automatically charged extra money as soon as excess data was used, which made her anxious about watching films or using internet radio while spending more hours than usual at home:

Ellen [Do] you listen to music on YouTube as well?

Heather Yeah, and on internet radio.

Ellen Okay, how's that going?

Heather All right. I have to be very careful when I do that because whereas I know that it does use up my data […] So I only do that once a week. […]

Ellen Do you have an amount of time that you know you can listen to the radio for?

Heather Not really. I just do it, I mainly listen to it for an hour and then give it a break and then another hour, I give it a break, like that. (Second interview, 1 May)

Heather replaced her pre‐lockdown strategy of travelling to her telecommunication provider's shop for updates on her data usage with a new strategy of self‐constraint (van Holstein et al., 2021). Her experience illustrates that, on top of the spatial constraints of stay‐at‐home orders, Heather was practising additional constraints that seemed financially necessary while she had not yet acquired a new way to monitor data usage. These circumstances aggravated the burden restrictions placed on Heather.

For Heather, the stress of lockdown intensified when a restaurant that she had visited became listed as an exposure site.

Heather That really shook me up. I actually – it made me go back to one of my bad habits.

Ellen Oh, what's that?

Heather I used to drink a lot of alcohol, but I only went to buy some alcohol, but I haven't – I didn't – I didn't – I actually didn't end up – end up drinking it. […] I feel that I'm going back to my bad habits because of this getting worried and worked up and staying at home all the time. (Third interview, 18 May)

When we spoke to Heather a week later, she described a state of alertness, and she described how diligence led to panic.

Heather: I've been having trouble sleeping, and when I get a headache or a sore throat I really panic. (Third interview, 22 May)

Heather's sense of panic about contracting the virus, combined with the stress of being forced to spend time isolated at home, created new risks in the form of temptation to relapse into potentially harmful practices. This vulnerability arose from an interplay of circumstances, including support providers' failure to deliver contracted services and embodied memories of coping with stress.

For another self‐advocate, the spatial reconfiguration of working from home created a new need to manage temptations and impulses. During and between lockdowns, leisure and workspaces collapsed into the space of the home, removing boundaries that were previously in place around shopping temptations. Luke described this new challenge:

Luke I must admit, thank god I'm earning money because you spend – when you're in isolation, you spend a lot of money.

Ellen Do you? How?

Luke Well, I've got an addiction. I read a lot of books, or audio books. So, I went a bit crazy on the website and bought about three or four or five books. […]

Ellen Did you use to buy things online before this started?

Luke No, because I sort of went – I sort of stayed away from it because it's a bit of a temptation. […] So, as soon as the isolation is over, I'm going to have to be careful what I buy. (First interview, 23 April)

In this interview, Luke explained that in his pre‐COVID work and shopping routine he avoided online shops and their temptations.

Luke We have breaks in meetings, so some days I'll go off and buy a book or buy something, or when I'm talking with my friends, I'm not really interested in what they say, I'm into books that I'm looking at. (First interview, 23 April)

On work breaks or while socialising with friends during the lockdown periods, temptations were continuous for Luke, because his previous efforts to eliminate online shopping were undermined by the constant presence of online shops in the merged home/workplace. This collapse of commercial space into the home created new vulnerabilities as risks and temptation creeped up in new ways that had not yet been accounted for with an adjusted set of practices.

For others, the multifunctional home presented challenges because it removed boundaries around emotions attached to work. Sue said about her self‐advocacy work:

[It] can get quite emotional, work, at the best of times. I always try to think […] I've just got to put my feelings aside, do my work and deal with them later, which meant I was dealing with them at home by myself which wasn't good. (Third interview, 19 May)

Sue's self‐advocacy work is focused on the rights of people with intellectual disability to be parents and to receive support in parenting. Sue's emotional investment in this work as a parent has made working from home especially stressful for her. Heather and Luke's stories show the importance of boundaries around spaces for work, leisure, and (self)care for people with intellectual disability. Sudden changes to these boundaries can create a disproportional strain on this group that finds expression in embodied manifestations of stress and boredom such as anxiety, sadness, and impulses.

### 5.4 Temporalities of vulnerability

Temporal dimensions of vulnerability played a significant role in shaping the self‐advocates' experiences of vulnerability. Past experiences of mistreatment sparked feelings of nervousness and limited the choices that seemed available to them for managing new circumstances. Heather's experience illustrates this well. When she was informed that she had visited an exposure site, she explored ways to avoid going to shops by using home delivery for her groceries. However, past experiences with online shopping made her not trust this option:

Heather: If I pick out a grocery item and they don't have it; they will replace it with something else that is probably two times more expensive. […]

Ellen: This has happened to you before? […]

Heather: Yes, and I wasn't – they wouldn't make it the same price. (Third interview, 18 May)

Heather explained that this experience occurred eight or nine years ago when she ordered an iPod from a retailer.

Ellen did you buy the iPod from the supermarket Heather?

Heather No, from JB Hi‐Fi.

Ellen Okay, so […] because of the experience of ordering with JB Hi‐Fi now you're nervous to order at the supermarket?

Heather Yep. Because I have a disability, they'll probably trick me into doing – buying something else similar and more expensive price.

Ellen So, instead you've been going to the supermarket yourself?

Heather Yeah, I would prefer to go myself. (Third interview, 18 May)

Based on past experience, Heather expects that people will take advantage of her because of her disability and has therefore delayed switching to online services. Heather's story shows how vulnerabilities that are created by the ‘crisis ordinariness’ of ableism (Berlant, 2011) feed into experiences of vulnerability during emergency, and can inform practices and decisions that in turn intensify vulnerability.

Vulnerability emerges here through restrictions that mandate people to stay at home combined with remote service systems that expose people with intellectual disability to risk of cost blow‐out. For Heather, going out to the shop creates risk of contracting the virus, while staying at home opens her up to the risk of being exploited online. Through considering these simultaneous risks and how they manifest in Heather's life, we can identify that vulnerabilities pre‐date the pandemic in ways that go beyond care needs. Rather, past experiences of poor treatment make Heather expect to be exploited or tricked, a risk she perceives as greater on online platforms where she is denied opportunities to intervene. Lingering experiences of ableism inform the way she balances these multiple concurring vulnerabilities. Additionally, low income and few savings increase the stakes of being exploited online, which makes the control that comes with her embodied presence in a shop weigh that much heavier.

The blurred boundaries between immediate and ongoing crisis also became evident in how some restrictions did *not* affect the self‐advocates. Some COVID‐19 restrictions were experienced as a mere continuation of existing restricted routines. Melbourne's night curfew, for example, was nothing new for Greg:

Ellen Do you feel like the evening curfew – has it changed anything for you?

Greg No, not at all because my parents don't allow me to drive at night anyway.

Ellen To go out at night?

Greg Yeah.

Ellen Okay. [Laughs] So you already had a curfew, Greg?

Greg [Laughs] Yeah. (Sixth interview, 10 August)

For Greg, the ongoing experience of being subjected to decisions made by others facilitated a sense of acceptance of lockdown restrictions.

In other instances, self‐advocates reported feeling nervous about getting into trouble with police or bystanders for breaching lockdown rules. They described past experiences of conflicts in which their account of events was not believed or taken seriously by people in positions of authority:

Heather No matter who I tell, I'm not taken seriously. I feel that when something does happen, like in an emergency, I still won't be taken seriously. (Fourth interview, 18 June)

When we asked Heather whether she minimised time spent outside the home, she said that she did not go out at all. For this same reason, Sue self‐imposed higher levels of restriction than required, to minimise the risk of violations:

Sue Sometimes I don't know what an hour is, so I just – the only thing I go out for is shopping or as I need things, I'll get them and then come straight back.

Ellen Do you mean that you lose track of time when you go out?

Sue Yeah, sometimes.

Ellen Okay, so that makes you nervous about overstaying?

Sue Yeah, yeah. (Sixth interview, 7 August)

Sue and Heather's stories illustrate that past experiences of being singled out have signalled to them that they find themselves in an ‘unwelcoming world and an unreliable environment’ (Berlant, 2011, p. 20). This motivated both Heather and Sue to self‐exclude and deepened their vulnerability to isolation. The focus on temporalities of vulnerability that a more‐than‐care lens adds to existing attention to the temporalities of care and caring (e.g., Power, 2019) demonstrates the emergence of vulnerabilities that pre‐date emergency‐induced needs for care and support.

## 6 TOWARD MORE‐THAN‐CARE IN GEOGRAPHIES OF VULNERABILITY

This paper has explored the experiences of self‐advocates with intellectual disability during pandemic lockdown. Their accounts of managing new tasks, circumstances, and risks suggested that the vulnerabilities that emerged during lockdowns were not a direct result of disability or insufficient or overly restrictive care, but the product of a suite of dynamic circumstances including ever‐changing rules and social norms, inaccessible services, disrupted strategic routines, and embodied experiences of living with intellectual disability. Contrasting with a narrower conceptualisation of vulnerability as a knowable, predictable, and preventable care need, we have explained how vulnerability must be expanded to register the inherent unpredictability of emergent vulnerabilities. With a few exceptions (Webber, 2013), geographers have been slow to acknowledge the dynamic nature of vulnerability, and the limitation this places on efforts to minimise adverse impacts on people labelled ‘vulnerable’ during crises. An explicit consideration of cognitive disability in processes of responding to crises has been less well articulated and has foremost been understood as a care gap that generates vulnerability when unmet. In response, this paper has sought to advance geographical thinking by highlighting how vulnerability emerges and how it is managed by people with intellectual disability with and beyond care.

In applying a more‐than‐care lens, our analysis indicated that people with intellectual disability can be active managers of vulnerabilities. Our findings therefore build on critiques of vulnerability as an outcome of the dependency of people with intellectual disability on care (Clough, 2017, 2021). The stories of lockdown presented in this paper show that self‐advocates' capacity to make decisions and direct support was entwined with their ongoing contact with colleagues, family, and friends, as both providers of care and as relations that at times inhibited decision‐making autonomy and capacity to self‐manage vulnerability. Furthermore, the temporalities and spatialities of such contact were radically disrupted during the pandemic. The findings demonstrate that, during crises, the boundaries between support with decision‐making and dependency can become blurred in relationships between some people with intellectual disability and their support workers, and this reveals the importance of considering cognitive ability in such entwinements. In building on Cluley and colleagues' observations about the assumed cognitively abled body in thinking about the embodiment of disability (Cluley et al., 2020), more‐than‐care points to new avenues for thinking about co‐corporeality that account for cognitive disability.

More‐than‐care expands the importance of embodiment for thinking about vulnerability. Geographers have engaged thoroughly with Butler's important observation that: ‘we all live with … vulnerability, a vulnerability to the other that is part of bodily life’ (cited in Harrison, 2008, p. 426). Geographers have eagerly adopted this understanding of vulnerability as an intrinsic aspect of corporeal existence (Harrison, 2008; Wiles, 2011). This paper expands those observations by pointing out that bodily susceptibility does not merely function as a source of vulnerability; embodiment also plays a crucial part in mediating vulnerabilities. Embodiment featured strongly in the self‐advocates' accounts of how they registered vulnerabilities and how they developed strategies for managing vulnerabilities. Embodiment also featured as a constitutive aspect of living with intellectual disability, which affirms the heterogeneous nature of what it means to live with intellectual disability. Cluley et al. (2020) recently argued that disability needs to be thought of as an ever‐changing phenomenon. The more‐than‐care framework builds on that argument by pointing out that disability changes during crisis as people adjust their routines, face new forms of hardship, and learn new skills. These changing conditions inform how a person with intellectual disability identifies and manages vulnerability.

The paper's findings reinvigorate discussions about the spatiality and temporality of vulnerability (Connon & Hall, 2021). Geographical work with people with intellectual disability underwrites the importance of interdependence for the achievement of independence and emphasises that caring and supportive relationships change the inclusiveness of places for people with intellectual disability (Power, 2010). This research has found that pre‐existing forms of exclusion and discrimination were essential aspects of the vulnerabilities that emerged during lockdown, as memories of injustice such as being exploited, told‐off, and dismissed meshed together with newly introduced rules to curtail possible coping strategies. The ways in which the self‐advocates experienced vulnerability were thus an intensification and expansion of the forms of disadvantage that they were already routinely experiencing and managing before the pandemic. This affirms observations that disability cannot be understood in isolation from its social context (Hall & Wilton, 2017). A focus on more‐than‐care enables analyses of vulnerability that decentre the disability category as a stand‐alone source of vulnerability as it considers individual needs and the social organisation of those needs in tandem.

Thinking about vulnerability with a more‐than‐care framework creates space to broaden current geographical thinking about responsibility for managing vulnerabilities. Understanding vulnerability as a slow emergency (Anderson et al., 2020), shaped in part by past experiences of discrimination and exclusion, creates an imperative to reduce vulnerabilities not only in relation to extreme disruptions such as pandemics or disasters but also everyday discriminatory practices. Furthermore, the framework creates a path for research on emergency support needs that starts from the ability of people with intellectual disability to manage vulnerabilities. The self‐advocates' accounts demonstrate that they rose to the occasion when personal responsibility became the dominant trope during lockdown, affirming the role of people with disability as actors in disaster prevention and response strategies (Connon & Hall, 2021). However, for people with intellectual disability this means that they become responsible for making the right decisions with minimal support. This comes at a price, including the self‐imposition of harsher restrictions than required. A more‐than‐care approach suggests that rather than avoid discussing the vulnerability of people with intellectual disability, geographers need to analyse how the agency to manage vulnerabilities is unevenly distributed due to ongoing injustices such as those of asymmetrical investment in infrastructures that hold up caring relationships and other strategies for getting by on which people with intellectual disability rely. While this paper has focused on people with relatively mild intellectual disability, the framework's attention to the person's agency, their ability, and how these shift under changing circumstances to give rise to new vulnerabilities also provides a fruitful avenue for future work with people with more severe disability.

At a policy level, more‐than‐care has the potential to recalibrate the objectives of personalised support mechanisms, such as the NDIS, that currently tend to focus on individual capacity‐building toward greater independence. In such systems, providers of support tend to understand disability support as a relatively predictable and plannable service for the benefit of a single person, in which support needs are balanced against an imperative for cost‐efficient service delivery. These market‐driven and individualised characteristics can fail to create just outcomes at times of emergency or crisis. This paper joins previous studies that have argued for a move away from a focus on people's individual capacity to respond to emergency and toward people's independence through interdependence (Power, 2010; Pyke & Wilton, 2020). This highlights the need for ongoing investment in adaptive support networks as new support needs emerge unexpectedly and existing networks of support can falter when spatial and temporal routines are disrupted. Furthermore, our research shows a need for support provision to be imagined in broader terms than paid disability support while acknowledging people with intellectual disability's agency as more than recipients of care. The self‐advocates made significant use of disability advocacy networks for interpreting complex information and for developing the new skills and resources required for navigating changed circumstances. This confirms previous findings that self‐advocacy groups offer people with intellectual disability collegiality and support, while organisations that accommodate self‐advocacy groups are structurally underfunded in disability support regimes that focus on the individual needs of care recipients (Anderson & Bigby, 2017) and it demonstrates the importance of investment in a broad suite of infrastructures for managing vulnerabilities in times of emergency.

## Data Availability

The data that support the findings of this study are available from the corresponding author upon reasonable request.
